# Measurements and computational fluid dynamics investigation of the indoor radon distribution in a typical naturally ventilated room

**DOI:** 10.1038/s41598-022-23642-7

**Published:** 2023-02-04

**Authors:** Mohammademad Adelikhah, Morteza Imani, Tibor Kovács

**Affiliations:** 1 Institute of Radiochemistry and Radioecology, University of Pannonia, Veszprém, 8200 Hungary; 2 Engineering Department, G.C, Shahid Beheshti University, P.O. Box: 1983963113, Tehran, Iran

**Keywords:** Environmental impact, Natural hazards

## Abstract

Based on the European Union Basic Safety Standards to protect people against exposure to ionizing radiation, establishing and addressing the reference levels for indoor radon concentrations is necessary. Therefore, the indoor radon concentration should be monitored and control in dwelling and workplaces. However, proper ventilation and sustainability are the major factors that influence how healthy the environment in a building is for its occupants. In this paper, the indoor radon distribution in a typical naturally ventilated room under two scenarios (when the door is closed and open) using the computational fluid dynamics (CFD) technique was studied. The CFD code ANSYS Fluent 2020 R1 based on the finite volume method was employed before the simulation results were compared with analytical calculations as well as passive and active measurements. The average radon concentration from the CFD simulation was found to be between 70.21 and 66.25 Bq m^−3^ under closed and open-door conditions, respectively, at the desired ventilation rate of 1 ACH (Air Changes per Hour). Moreover, the highest concentrations of radon were measured close to the floor and the lowest values were recorded near to the inlet, resulting in the airflow velocity profile. The simulation results were in good agreement with the maxima of 19% and 7% compared to analytical calculations at different indoor air velocities in the open- and closed-door scenarios, respectively. The measured radon concentrations obtained by the active measurements also fitted well with the CFD results, for example, with a relative standard deviation of around 7% and 2% when measured by AlphaGUARD and RAD7 monitors at a height of 1.0 m above the ground in the open-door scenario. From the simulation results, the effective dose received by an individual from the indoor air of the workplace was also calculated.

## Introduction

Over the last few decades, a significant degree of concern worldwide has been raised about the health risks following exposure to radon and its daughters. On the one hand, people are spending approximately 80% of their life in confined and closed places, i.e., in homes and offices, but on the other hand, almost 50% of the natural background radiation dose to humans originates from the inhalation of these gases and their airborne progenies^1,2^. Radon is a radioactive gas. It is the decay product of ^226^Ra having half-life of 3.84 days and an alpha particle emitting inert radionuclide (5.49 MeV). It is naturally occurring nuclides and considered as the hazardous gas for human living environment as alpha particles can cause substantial damage to a cell. The decay products of radon may be deposited in the lung tissues in the heterogeneous pattern^1^, and known as the second leading source of lung cancer after smoking^2^. Therefore, knowing the behavior and distribution of indoor radon, that is, the exact levels of radon at different points and areas, especially in breathing zones in residential buildings, is essential for the purpose of dose assessment^3–5^. Apart from this, indoor radon exposure has been estimated to account for approximately 9% of all lung cancer deaths and 2% of all cancer deaths in Europe^6^. In Hungary, regarding the new updated standards of the European Union Basic Safety Standards^7^, it is recommended that the annual average indoor radon concentration should not be higher than 300 Bq m^−3^ in dwellings or workplaces^7^ and the member countries should prepare and continuously review a Radon Action Plan to reduce the lung cancer risk of the radon exposure. Therefore, the indoor radon concentration should be monitored and control in dwelling and workplaces.

Since radon comes from the natural decay of uranium that is found in nearly all soils, it typically moves up through the ground to the air above and into your home through cracks and other holes in the foundation. Your home traps radon inside, where it can build up. Any home may have a radon problem. This means new and old homes, well-sealed and drafty homes, and homes with or without basements. Radon from soil gas is the main cause of radon problems. Sometimes radon enters the home through well water. In many of homes, the building materials can give off radon, too. However, building materials rarely cause radon problems by themselves. Hence, being a noble gas, radon easily gets released from the source term to the pores (emanation) and subsequently from the pores to the outside environment (exhalation).

A number of techniques have been used to measure the radon concentrations and their decay products in the environment including Active and Passive methods. The devices and methods may vary over a wide range of grab sampling, time-integrated sampling (short-term and long-term) and continuums sampling (known as real-time radon monitoring). However, radon measure devices are also classified differently based on their used method of radon monitoring in addition to other classification, i.e., electrostatic collection of decay products (RAD7, Tesla TSR2, EQF3220), ionizing chamber device (AlphaGUARD), photo-multiplier counter and scintillation (Liquid scintillation or scintillation cell), radon absorption (active charcoal), etched track detectors (CR-39, LR115). We have to decide what technique to use on the basic of the feasibility and cost of the measurement as well as accuracy and applicability of the technique.

In case of standard problems for measuring of indoor radon concentrations, indeed, radon measurements in homes are easy to perform, but need to be based on standardized (e.g., national) protocols to ensure accurate and consistent measurements. They do not address all technical aspects of measurement device technology, quality assurance or techniques to specifically identify radon sources such as radon in water supplies, building materials or relative to the possession and handling of radioactive materials. In addition, high variation of indoor radon makes short-term measurements unreliable for most applications. Another problem is related to the type of detector which should be carefully selected since it influences the cost of measurement per dwelling and therefore the cost of a radon program on a national level.

In confined areas, a vital factor with regard to human health, indoor air quality and energy efficiency is the ventilation rate since the degree of exposure can be significant, especially in buildings with poor ventilation systems where radon gas, which is heavier than air, can easily accumulate and reach lethal activity concentrations in terms of human health. It is worth mentioning that ventilation does not directly affect occupant health, but the rate of ventilation affects indoor air pollutant concentrations that, in turn, modify the occupants’ health. Previous studies have reported that indoor radon and thoron concentrations are related to environmental meteorological parameters and building ventilation conditions by applying numerical and experimental methods as well as different pieces of computational fluid dynamics (CFD) software as analytical and powerful tools^8–14^. For instance, Zhou et al. applied the finite difference method to derive discrete equations before linking them to the commercial FU-JITSU/a-FLOW code to study the concentrations and their distributions of ^222^Rn and ^220^Rn as well as their progenies in a model room^8^. Rabi et al. implemented a ^222^Rn distribution inside a typical Moroccan room using Fortran software^12^. Chauhan et al. and Agarwal et al. in this regard also used the software Fluidyn MP based on the Finite Volume Method (FVM)^11,14^. As a result, appropriate ventilation could reduce indoor pollution due to radon exhalation from the ground or from contaminated building materials.

Radon, after the release from the walls and floor, gets distributed in indoor environment. Understanding its distribution is important to predict the spatial and temporal variations of levels which can ultimately be used for dose calculations and exposure control research. As the time spent indoor is large enough; understanding, prediction and measurement of indoor radon distribution becomes quite important. In addition, the concentration level and spatial distribution of radon may be affected by the dwelling's ventilation conditions. The main purpose of this study is to estimate the indoor radon distribution in a typical naturally ventilated test room under the following two scenarios: closed- and open-door conditions. The need of the study is the prediction of activity level and to study the effect of natural ventilation on indoor radon. Therefore, the inlet velocity was calculated on the basis of the ventilation rate. Hence, based on the American Society of Heating, Refrigerating and Air-Conditioning Engineers (ASHRAE) Standard 62.1, ventilation standards in buildings are suggested to meet indoor air quality regulations which take into account the volume or area and the number of inhabitants^15^. Furthermore, Yoshino et al. reported that the minimum air exchange rate (it is the number of times that the total air volume in a room or space is completely removed and replaced in an hour) in the majority of European countries is 1 h^−1^, which is also in accordance with Japanese regulations concerning air ex-change rates in buildings^16^. By taking into consideration the aforementioned criteria, a different range of ventilation rates from 0.3 to 4.3 h^−1^ (also reported by Zhou et al.^8^ and Agarwal et al.^14^) was considered and assessed in the current study. The radon source term as a key input parameter in the CFD software (ANSYS Fluent 2020 R1 based on the FVM) has also been measured by an accumulation chamber technique for samples of cement (comprehensively described in Kocsis et al.^17^ and Shahrokhi et al.^18^) before the simulations took into account the room geometry. To validate the simulation results, two pairs of common radon monitoring methods, namely NRPB and Raduet (as passive methods based on CR-39) as well as AlphaGUARD and RAD7 (as active methods), were also used to measure the indoor radon concentration under the aforementioned scenarios at various locations throughout the room.

## Models and computational methods

### Geometric model

**Figure 1:**
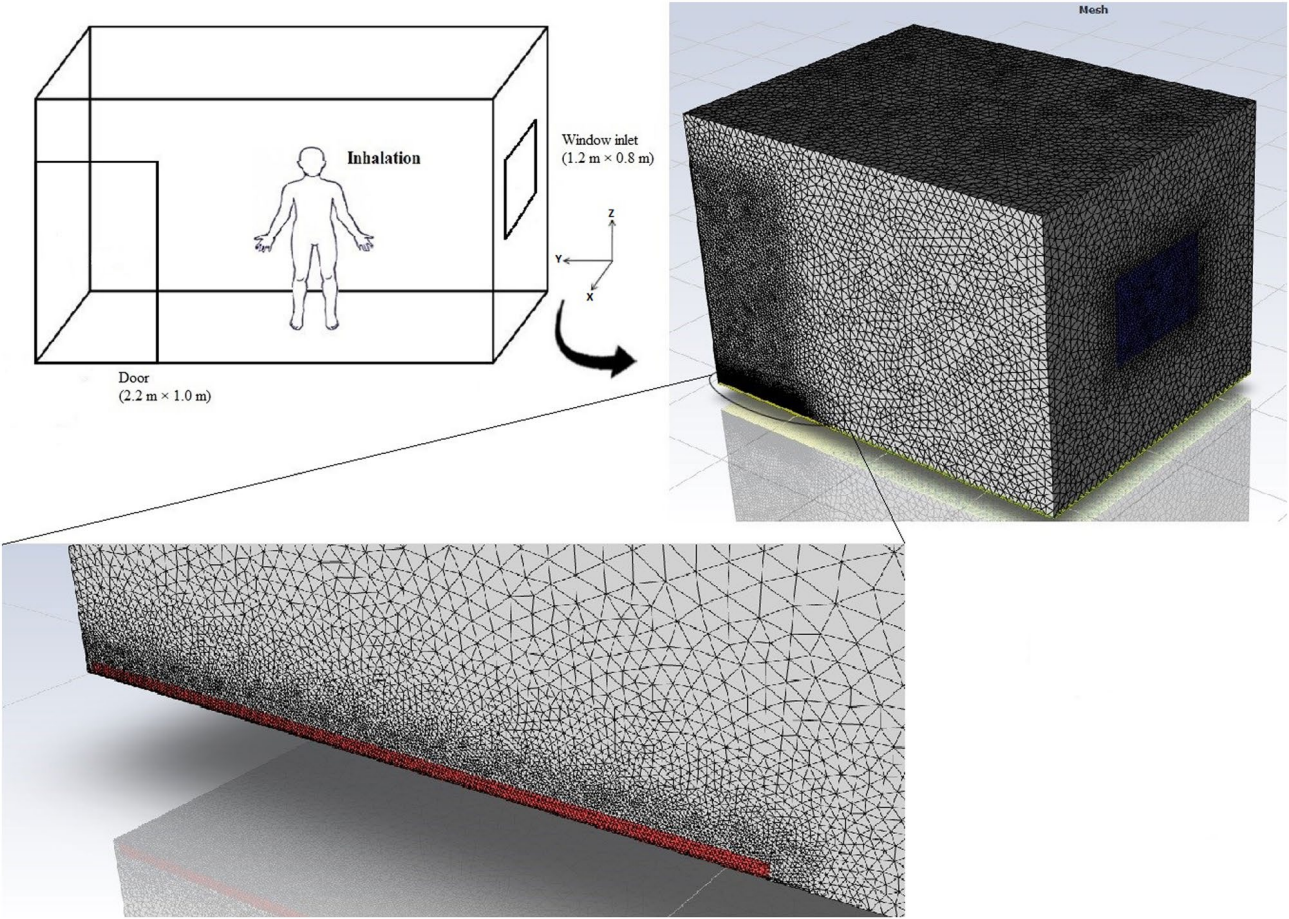
Schematic diagram of the model room with its meshing.

**Figure 2:**
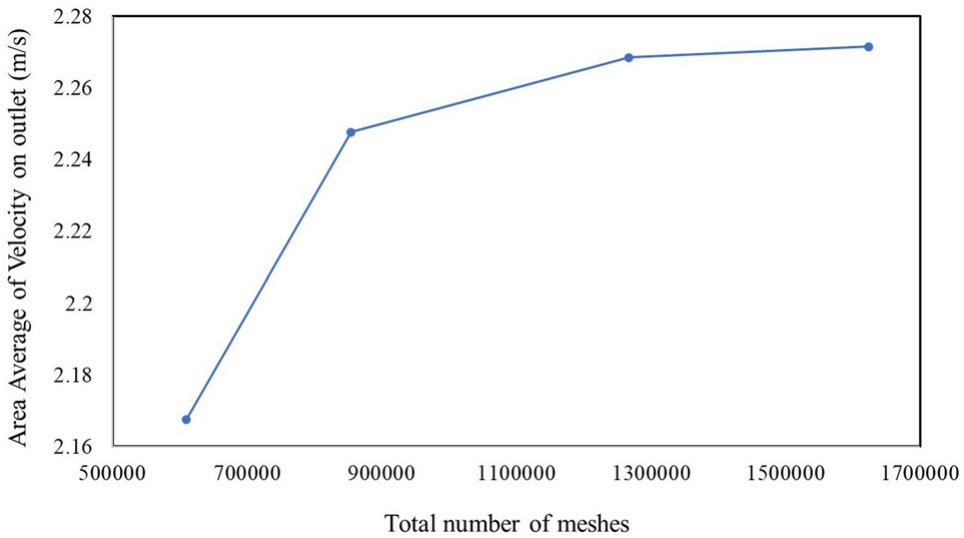
Convergence study of the model.

The geometric model considered in this study was based on the typical size of a room at the Institute of Radiochemistry and Radioecology at the University of Pannonia in Hungary (Fig. 1). The overall computational dimensions of the room geometric model were 3.0 m (W) × 4.0 m (L) × 2.8 m (H) along the X, Y and Z-axes, respectively, including one window (1.2 m × 0.8 m) in the middle of the wall on the right-hand side, which faces the outdoor environment, and a door (2.2 × 1.0 m) on the left-hand side of the front wall. Furthermore, unstructured triangular meshes were used for ANSYS meshing due to their simplicity and degree of accuracy which is accessible for our simple geometry. In addition, the unstructured mesh has a high-efficiency mesh distribution, which permits creation of fewer cells than a structured one^19^. The convergence study has been performed for the model and the average area velocity at the outlet has been considered as a basis. In this study, the close-door configuration with Ach 4.3 (Air changes per hour) (h^−1^) simulated for four different types of the meshing. The convergence of the model is shown in Fig. 2; consequently, the total number 1,267,543 cells with minimum volume of 2.3 × 10^–9^ m^3^ has been used for the analysis.

### Numerical modelling approach, boundary conditions and Parameters

CFD computer codes solve the set of conservation of mass, energy and momentum equations to specify the fluid flow and related phenomena. By discretizing and linearizing equations as well as under the relevant boundary conditions, the computational domain is defined. In this study, some assumptions are considered: (A) air enters the room from the outer environment through the window (inlet) and leaves the room through the door (outlet); (B) continuous and incompressible air flow inside the room; and (C) homogeneous indoor temperature distribution. Therefore, the steady-state indoor flow field could be expressed by continuity and conservation of momentum equations as follows, respectively^8,11^:

1 $$\rho \left( {\nabla .U_{i} } \right) = 0$$

2 $$\rho \left( {\frac{{\partial U_{i} }}{\partial t} + \nabla .\left( {U_{j} U_{i} } \right)} \right) = - \nabla .P + \nabla .\left( {\mu_{e} \nabla U_{i} } \right) + S$$

In the above equations, *U*_*i*_ and *U*_*j*_ denote the velocity vectors (m s^−1^) (i, j are the indices representing the velocity components); *P* represents the pressure (N m^−2^); *μ*_*e*_ = (*μ* + *μ*_*t*_) stands for the effective viscosity (N s m^−2^), where *μ* and *μ*_*t*_ refer to the dynamic and turbulent viscosities, respectively; *ρ* is the density (kg m^−3^) and *S* is radon source term (Bq m^−3^ s^−1^). Moreover, in order to simulate the dispersion of radon inside the room, the advection–diffusion equation is also applied:

3 $$\frac{\partial C}{{\partial t}} = S + \nabla .\left( {D\nabla C} \right) - \nabla .\left( {UC} \right) - \lambda C$$

where *C* represents the radon concentration in the room (Bq m^−3^), *S* stands for the radon source term (Bq m^−3^ s^−1^), *D* denotes the radon diffusion coefficient in air (1.2 × 10^–5^ m^2^ s^−1^), *U* refers to the mean air flow velocity (m s^-1^) and *λ* is the decay constant of radon (2.1 × 10^–6^ s^−1^).

On the other hand, since creating an appropriate model and characterizing suitable boundary conditions both play a key role in employing CFD techniques, some major boundary conditions and parameters are applied in this study:

- The inlet air velocity was calculated by taking into account the ACH value. The air velocity in terms of the inlet boundary condition (window) corresponding to the different ventilation rates and the ventilation area was calculated by the following equation^8,11^:
  4 $$V = \frac{{Ach \times V_{room} }}{{A_{vent} }}$$

where *V*_*room*_ and *A*_*vent*_ denote the volume of the room, which was assumed to be 33.6 m^3^, and the ventilation area (window area = 1.2 m × 0.8 m), respectively. Normally, 1 ACH is adequate to meet ventilation requirements. In this study the inlet air velocity was calculated to be approximately 0.01 m s^−1^ to validate the CFD simulation results by following passive and active methods.

- For the room parameters and inlet velocities, since the calculated Reynolds numbers were found to be greater than the 2000 when ACH = 1 h^−1^ and higher (turbulent regimes), the standard k-ε model, which has been used by many scholars^8,10,11^, was used to incorporate the effect of turbulence on the flow field given that it is capable of describing the investigated phenomenon.
- Another major input parameter is surface radon exhalation rates. Average surface radon exhalation rates for cement samples were measured to be 3.1 ± 0.1 (Bq m^−2^ h^−1^) according to a closed accumulation chamber technique using a professional AlphaGUARD PQ2000 PRO, which has been outlined in detail by Kocsis et al.^17^. Furthermore, Porestendorfer has summed up the others surveys and reported the typical range of surface radon exhalation rates for building materials used in different countries which fall within the range of 0.36–10.8 Bq m^−2^ h^−1^^20^. The values reported in this study are also in line with these ranges. Consequently, the rate of radon generation (Bq m^−3^ h^−1^), as an input parameter in the CFD code, can be calculated from Eq. 5:
  5 $$G = \frac{{\mathop \sum \nolimits_{i = 1}^{3} E_{i} \times A_{i} }}{{V_{room} }}$$
  where *i* = 1, 2 and 3 denote the wall, floor and ceiling of the room, respectively, while *E*_*i*_ (Bq m^-2^ h^-1^) and *A*_*i*_ (m^2^) represent the radon exhalation rate and surface area, respectively.
- In this study, the average outdoor radon concentration was also measured to be approximately 10 Bq m^−3^ before being converted and used as an input in the CFD code.
- In this simulation, the convergence criteria is defined as the maximum relative difference between two consecutive iteration must be less than 10^–6^.

In Table 1, a list of all boundary conditions for each surface of the model is presented. By selecting the species transport model in ANSYS Fluent, all volumetric species, including radon, air and water vapor, were defined. For modelling humidity, water vapor content would be defined in the model as a species. The other materials considered in the model are lightweight concrete for floors, dense concrete for walls, window materials and basic door materials. Subsequently, simulations were run until convergent results were obtained at different ventilation rates. Finally, software solved all the relevant equations by the coupled scheme with second order of discretization, and the mass fraction of radon was predicted before being converted into an activity concentration (Bq m^−3^).

**Table 1:** Boundary conditions of each surface of the room.

Surface	Boundary condition	
	Open	Close
Door	Outlet pressure (Atm)	No slip
Window	Inlet velocity	Inlet velocity
Walls	No slip	No slip
Floor	No slip	No slip
Ceiling	No slip	No slip
Bottom of the door	Outlet pressure (Atm)	Outlet pressure (Atm)

### Analytical calculation

In a ventilated room, the radon diffusion coefficient is disregarded and the radon transport equation or radon concentration in a building or room with volume V is described ^21^ as the following:

6 $$C_{i} \left( t \right) = C_{0} e^{ - \lambda t} + \frac{EA}{{V\lambda }} \left( {1 - e^{ - \lambda t} } \right)$$

where *C*_*i*_ is the indoor radon concentration (Bq m^−3^) at time t (h), *C*_0_ is either the initial radon content at *t* = 0 (h) or the outdoor radon concentration, λ is the total radon decay rate and ventilation rate (λ = λ_*R*n_ + λ_*V*_) in h^-1^, *E* (Bq m^−2^ h^−1^) is the radon flux or radon exhalation rate from the soil or building material, *A* is the exhalation surface area (m^2^) and *V* is volume (m^3^) of the house.

### Passive and active indoor radon measurements

In this survey, Raduet and NRPB detectors which are solid-state nuclear track detectors (SSNTDs) were used to make the passive measurements. A CR-39 detector, which is used to detect alpha particles emitted from radon and their progenies, is placed at the bottom of each chamber with sticky clay. The detectors were hung on three horizontal planes in the investigated test room for 45 days, which were defined as Z = 0.2, 1.0 and 1.8 m above the ground as well as positioned at least 20 cm away from any of the wall surfaces. The plane at a height of 1.0 m above the ground was regarded as the breathing zone for a standing adult. After exposure, all the detectors were washed with distilled water and dried before being chemically etched. The etching conditions for CR-39 were as follows: a solution of 6.0 M NaOH at a temperature of 90 °C for 3 h. The track densities were counted using an optical transmission microscope and image analysis software^4^. The calibration factors were also determined as a result of exposure tests in radon calibration chambers at the Institute of Radiochemistry and Radioecology at the University of Pannonia in Hungary as comprehensively described by Adelikhah et al.^3^. In the case of the active measurements, an AlphaGUARD PQ2000 PRO monitor and RAD7 radon-thoron detector made by DURRIDGE, USA were also used to continuously measure the radon concentration at different positions in the room in the open- and closed-door scenarios. The AlphaGUARD PQ2000 PRO monitor has an alpha spectrometer using an ionization chamber. The device was used in the diffusion mode over 60-min cycles for 24 h and the resulting time-averaged value was assumed to be the radon concentration. The RAD7 detector was set to a cycle time of 1 day mode. Furthermore, the active detectors were equipped with integrated sensors to measure the temperature, relative humidity and atmospheric pressure.

### Annual radon effective dose rate

In order to estimate the annual radon effective dose rate (AED) originating from the inhalation of indoor radon, the following equation is used^1^:

7 $$AED = C_{Rn} \times F \times t \times K$$

where *AED* stands for the annual radon effective dose rate from exposure to radon (mSv yr^−1^), *C*_*Rn*_ denotes for the average radon concentrations in the room (Bq m^−3^), *F* represents the indoor equilibrium factor for radon of 0.4 which was provided by United Nations Scientific Committee on the Effects of Atomic Radiation (UNSCEAR) in 2000, and *t* refers to the number of hours spent inside annually (2000 h based on the spent time by the staff). Furthermore, *K* denotes the radon dose conversion factor recommended by the International Commission on Radiological Protection (ICRP) Publication 115 of 12 nSv per unit of integrated radon concentration (Bq h m^−3^)^22^.

## Results and discussion

The CFD technique based on the finite volume method was used to predict, visualize and calculate the radon distribution and concentration inside the room as well as the mixture of indoor radon-air flow. Moreover, by using the obtained average indoor radon concentrations, the effective dose rate the staff are exposed to was also estimated.

### CFD simulations results

**Figure 3:**
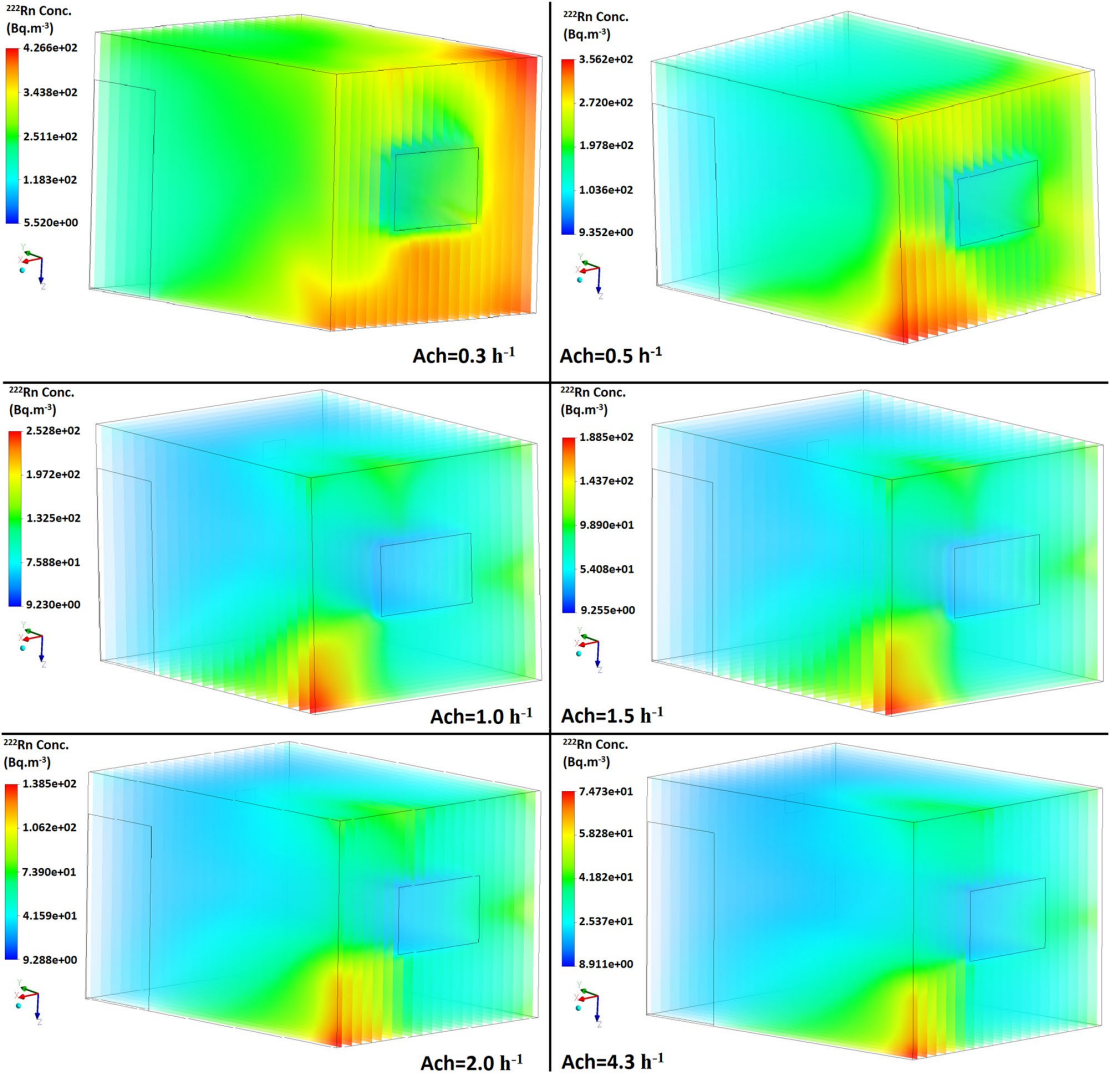
Distribution of the radon concentration in the closed-door scenario at different ventilation rates.

**Figure 4:**
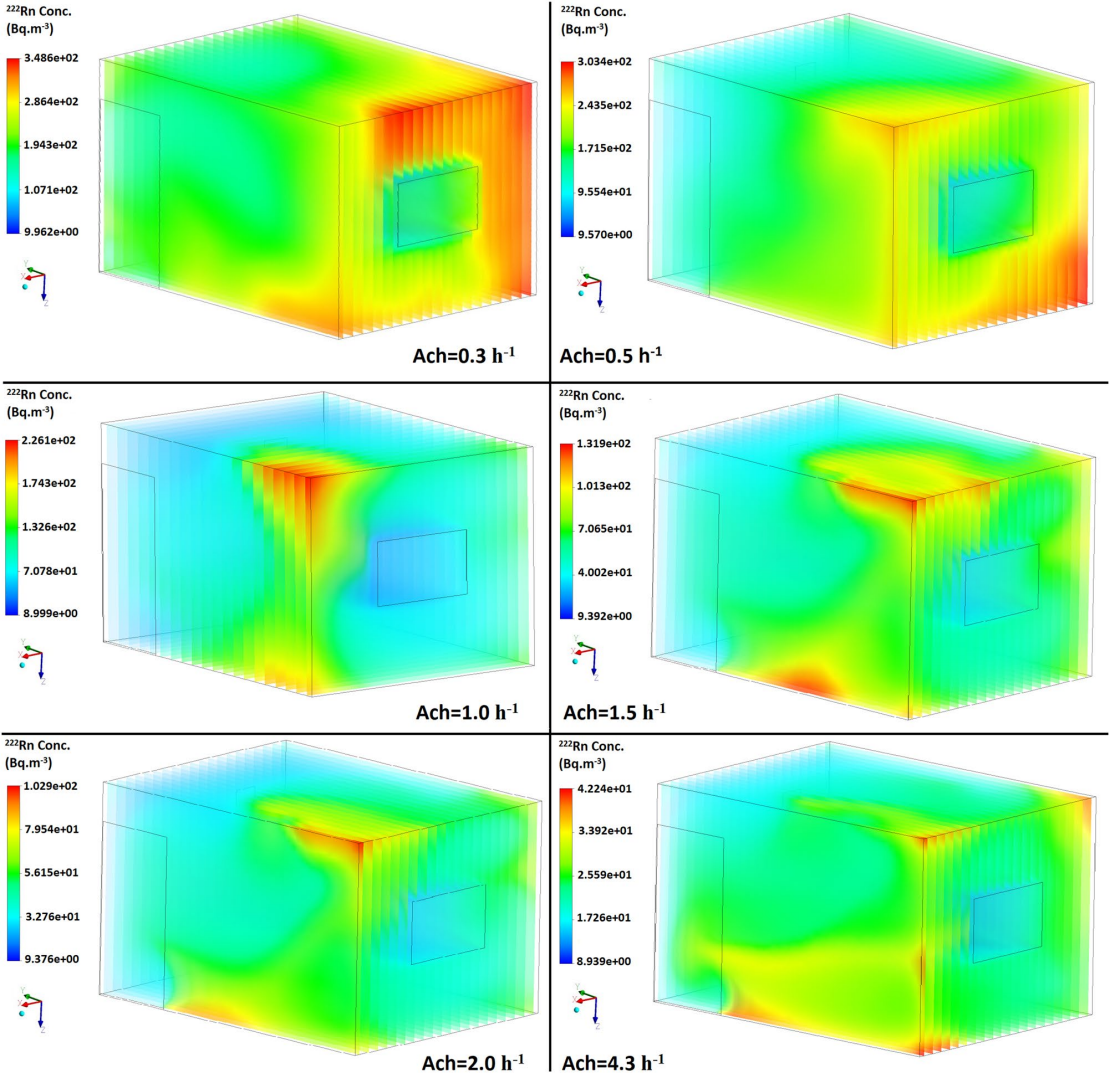
Distribution of the radon concentration in the open-door scenario at different ventilation rates.

By setting up the input parameters in the CFD code, the contours of the radon distribution at different air flow velocities in both aforementioned scenarios were simulated and are illustrated in Figs. 3 and 4. From the CFD results, it can be seen that as a result of the air flow velocity through the door and window, the radon gas concentration led towards the center of the room. Accordingly, the radon accumulated nearer to the surface of the left-hand corner of the room when the ACH was increased to 4.3 h^−1^. The ventilation profiles revealed that the indoor radon distribution was not uniform. By assuming that ACH = 1 h^−1^ in order to comply with ventilation requirements of buildings, the radon concentration in the middle of the room (in both scenarios) was low, moreover, the average radon concentration according to the CFD simulation was found to be 70.21 and 66.25 (Bq m^−3^) for the closed- and open-door scenarios, respectively.

**Figure 5:**
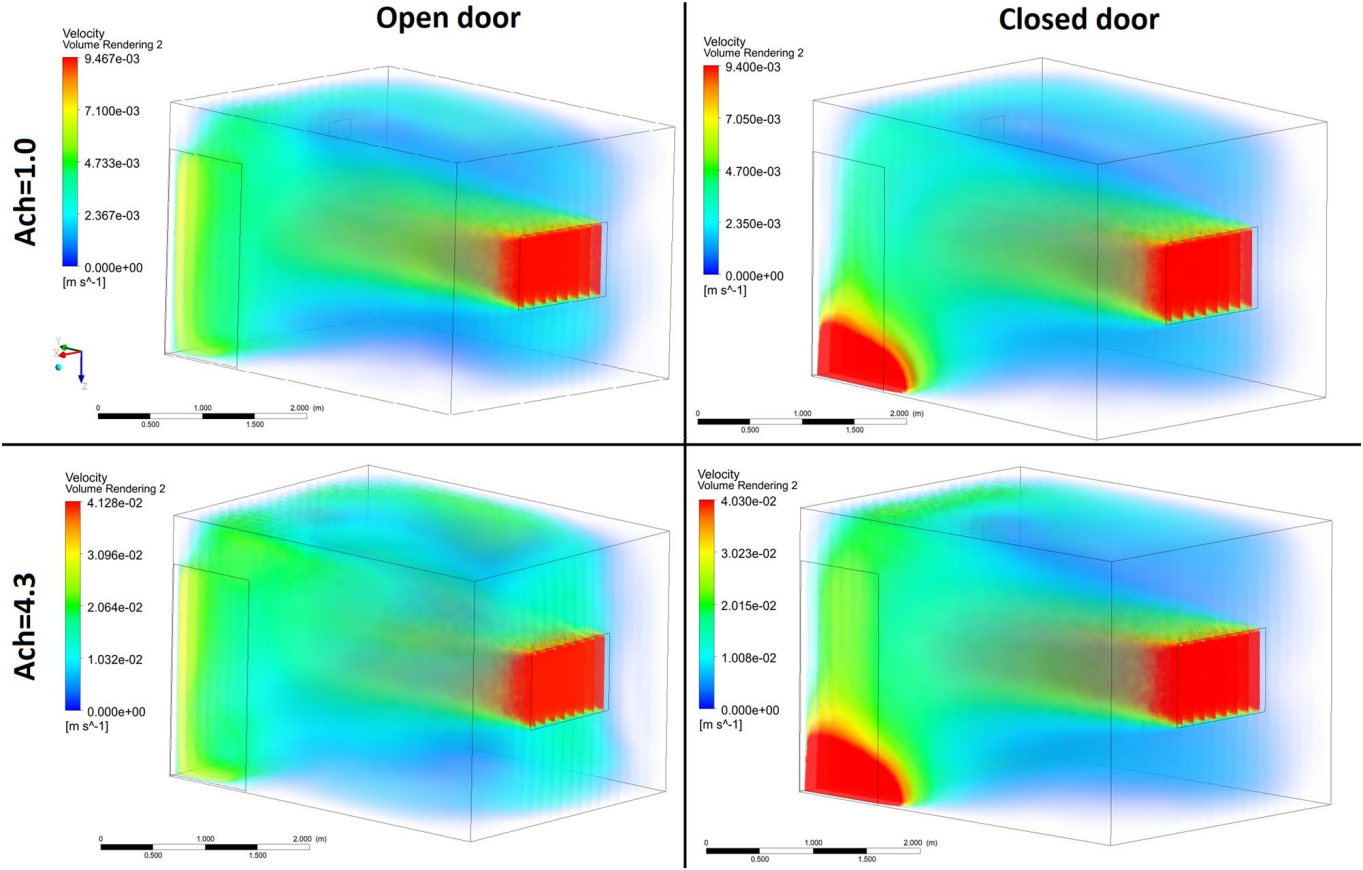
Velocity distribution in the modelled test room in both scenarios at 2 different ventilation rates.

**Figure 6:**
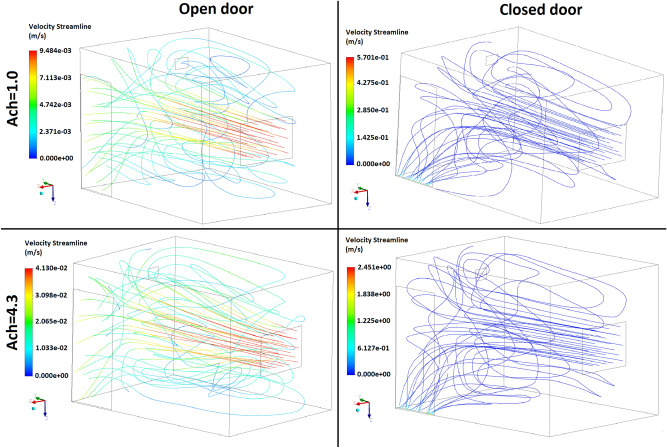
Streamlines in the modelled test room in both scenarios at 2 different ventilation rates.

Based on Figs. 3 and 4, the highest concentrations of radon were recorded close to the floor and upper wall around the inlet which are reduced by increasing the air exchange rate, while the lowest values were observed near to the inlet and front wall. These results are due to the air velocity profile (m s^-1^) in the room shown in Figs. 5 and 6, which was simulated in both scenarios at two different air exchange rates of ACH = 1 and 4.3 h^−1^ to comply with ventilation requirements and to compare with others studies, respectively. Increase in flow-rate generates higher turbulent kinetic energy in the higher velocity gradient region, thus increasing the turbulent intensity at some places. Spread of higher turbulent intensities increases with enhancing flow rates, and affects the mixing patterns and concentration inside the room. Moreover, moving up from the floor enhances the flow mixing, and affects the source contribution simultaneously. In order to compare the CFD results with other studies, Visnuprasad et al.^23^ and Zhuo et al.^8^ assumed ACH = 4.3 h^−1^ in the open-door scenario and the average indoor radon concentrations in their studies were reported to be 29 and 15 Bq m^-3^, respectively, while in this study it was simulated to be 20 Bq m^−3^, the results of which are given in Table 1. The average indoor radon concentration reported by Rabi et al.^12^ was 49 Bq m^−3^ which assumed that ACH = 1 h^−1^, in the closed-door scenario, while in this study the corresponding value was around 70 Bq m^−3^.

In this survey, the average indoor radon concentration according to CFD calculations represents the annual average radon concentration. Finally, according to the simulation results, the corresponding annual effective dose from the inhalation of radon when ACH = 1 h^−1^ was calculated as 0.68 and 0.64 mSv yr^−1^ in the closed and open-door scenarios, respectively. These annual effective doses are less than the limit recommended by ICRP of 3–10 mSv yr^-1^^24^. However, as simulated and shown in Figs. 2 and 3, it could be inferred that due to the poor ventilation and air velocity profile at some locations in the test room, e.g. close to the floor in the inlet, radon gas can accumulate more so the risk of exposure the staff are subjected to would be higher. Therefore, at this location, the corresponding dose received by the staff 1 m above the floor and when ACH = 1 h^−1^ could be approximately 1.63 and 1.48 mSv yr^−1^ in the closed and open-door scenarios, respectively, which are also less than the range recommended by the ICRP of 3–10 mSv yr^-1^.

**Figure 7:**
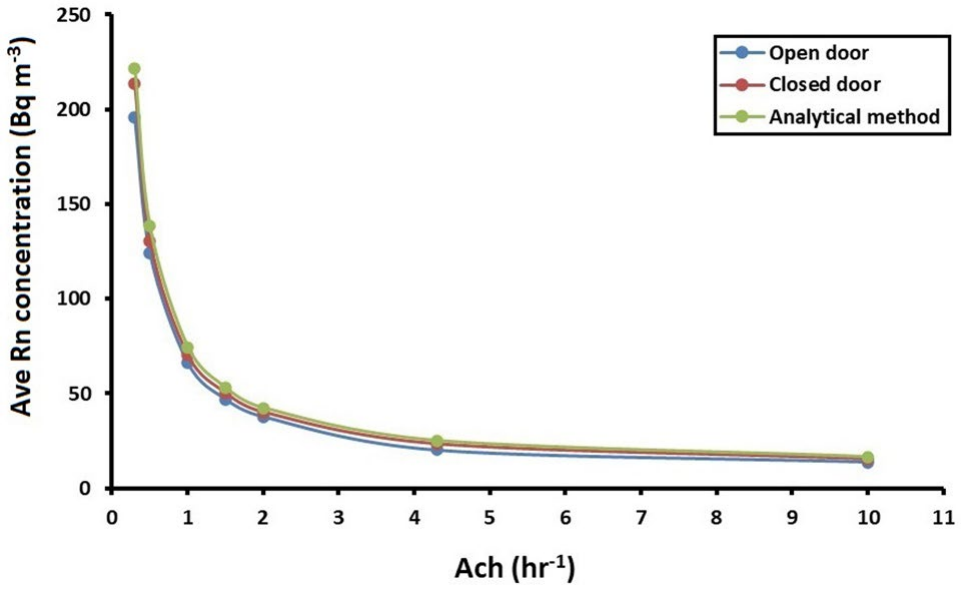
Averaged indoor radon concentrations in the room against the air exchange rate according to outputs of the CFD model.

In Table 2, the results of the analytical calculation and CFD simulation are compared. By computing the percentage difference between the estimated results according to ANSYS-Fluent and the analytical calculations at each ventilation rate, the maximum difference was found to be 19% when ACH = 4.3 h^−1^ in the open-door scenario. At the desired air exchange rate of 1 h^−1^, the difference was also found to be approximately 11% and 5% in the open- and closed-door scenarios, respectively. As is evident from Table 1, the different ventilation rates have distinct effects on the indoor radon concentration in the test room, which is also illustrated in Fig. 7. The simulation results indicate that the air flow pattern within the room is an important function with regard to the distribution of the indoor radon concentration. Moreover, it is noteworthy that the indoor radon concentration varies depending on the size of the room, radon exhalation from building materials and the air exchange rate.

**Table 2:** Variation in the volume averaged radon concentration (Bq m^-3^) in the room at different ventilation rates compared with the analytical calculation.

ACH (h^−1^)	Numerical simulation (Bqm^−3^)		Analytical method (Bqm^−3^)	Difference (%)	
	Open door	Closed door		With open door	With closed door
0.3	195.65	213.70	221.60	11.71	3.56
0.5	124.07	130.37	138.22	10.23	5.68
1	66.25	70.21	74.59	11.17	5.87
1.5	47.07	50.33	53.17	11.46	5.34
2	37.76	40.10	42.42	10.97	5.47
4.3	20.17	23.32	25.11	19.67	7.11

### Effect of relative humidity on indoor radon concentration

**Figure 8:**
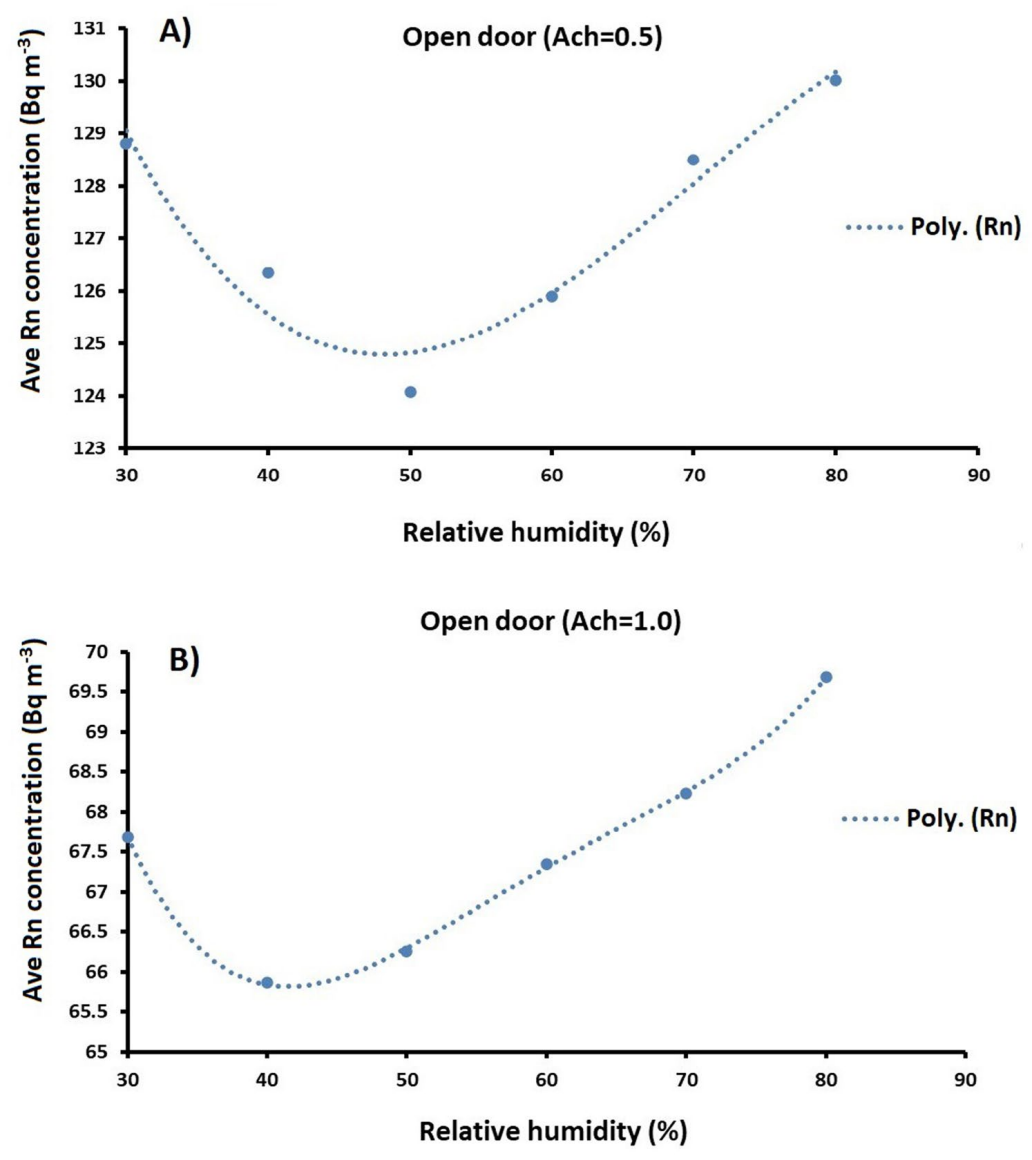
Effect of the relative humidity (%) on the average indoor radon concentration (Bq m^−3^) in the open-door scenario at two different ventilation rates according to the output of the CFD model.

Factors that affect the radon concentration in the room include building materials, ventilation rate, wind effect, temperature difference between inside and outside the room as well as the indoor air humidity. Regarding indoor air humidity, a negative correlation is observed between this parameter and the ventilation rate^25^. In this study, different values of the relative humidity (30%, 40%, 50%, 60%, 70% and 80%) are considered in the CFD code to explore the influence of the relative humidity on the indoor radon concentration. The temperature and air exchange rate were set at 24 °C and 0.5 to 1 h^−1^, respectively. By applying these assumptions and running the code, the results of the CFD model as well as the relationship between the relative humidity and average indoor radon concentrations (Bq m^−3^) in the room were plotted in Fig. 8A, B. This was simulated at two different air exchange rates to present the effect of the relative humidity on the indoor radon concentration. Accordingly, it can be seen that by increasing the relative humidity from 30 to 50%, the average indoor radon concentration was reduced by approximately 5% and then started to rise by increasing the relative humidity. Therefore, this clearly indicates that the relative humidity influences both the radon concentration and distribution.

### Validation of the simulations results with Passive and Active indoor radon measurements

The measured values of the radon concentration according to active and passive methods were compared with CFD predictions at the same points. The comparisons are presented in Tables 3 and 4 in the open- and closed-door scenarios, respectively. Accordingly, the average indoor radon concentrations measured by the AlphaGUARD and RAD7 detectors, for instance, at a height of 1.0 m above the ground in the open-door scenario (regarded as the breathing zone for a standing adult) were 77 Bqm^−3^ and 81 Bqm^−3^, respectively, which exhibit a relative deviation of approximately 7% and 2% (Relative deviation = $(\left(\left|\mathrm{Measurement}-\mathrm{CFD prediction}\right|\right)$/CFD prediction). Regarding the passive measurements according to the Raduet and NRPB detectors, the corresponding average indoor radon concentrations were measured as 68 and 64 Bqm^−3^, respectively with corresponding relative deviations of approximately 17% and 23%. However, in the closed-door scenario, the corresponding relative deviation was higher. Furthermore, the highest relative deviation of 39% was measured by the NRPB detectors from 20 cm above the ground in the closed-door scenario. As a result, it can be observed that both experimental results and simulations somehow yielded a similar trend, that is, the radon concentration reduced as the distance from the ground increased. Furthermore, based on the deviations, the average indoor radon concentrations predicted from the CFD code were seen to be closer to the experimental values with the exception of point A in both scenarios due to the poor air circulation resulting in the accumulation of radon at that point. Correspondingly, the results of the CFD simulations are in good agreement with the experimental measurements.

**Table 3:** Comparison of the CFD results (Bq m^−3^) with those obtained by active and passive measurement techniques at different levels in the open-door scenario when ACH = 1 (h^−1^).

Z (cm)	Device	(X,Y) coordinate (Bqm^−3^)				Center (Bqm^-3^)	Average (Bqm^−3^)
		A (2.9,0.1)	B (0.1,0.1)	C (0.1,3.9)	D (2.9,3.9)		
20	Alphagaurd	119	112	81	58	84	91
	RAD7	110	103	84	51	89	87
	Raduet	88	73	66	62	–	72
	NRPB	74	60	69	55	–	65
	Simulation	163	115	65	46	76	93
100	Alphagaurd	92	102	67	55	70	77
	RAD7	109	93	71	58	74	81
	Raduet	66	83	61	63	–	68
	NRPB	71	69	59	56	–	64
	Simulation	154	98	50	52	57	82
180	Alphagaurd	–	–	–	–	–	–
	RAD7	–	–	–	–	–	–
	Raduet	65	68	55	60	–	62
	NRPB	63	58	60	55	–	59
	Simulation	199	117	49	50	61	95

**Table 4:** Comparison of the CFD results (Bq m^−3^) with those obtained by active and passive measurement techniques at different levels in the closed-door scenario when ACH = 1 (h^−1^).

Z (cm)	Device	(X,Y) coordinate (Bqm^−3^)				Center (Bqm^−3^)	Average (Bqm^−3^)
		A (2.9, 0.1)	B (0.1, 0.1)	C (0.1, 3.9)	D (2.9, 3.9)		
20	Alphagaurd	93	109	74	63	91.5	86
	RAD7	120	99	82	66	95.8	92
	Raduet	88	79	58	65	–	73
	NRPB	77	65	72	59	–	68
	Simulation	224.43	133.41	68.57	54.42	82.35	112
100	Alphagaurd	110	108	61	63	84.6	85
	RAD7	119	105	68	61	77.1	86
	Raduet	89	92	66	74	–	80
	NRPB	76	66	58	63	–	65
	Simulation	170.7	140.18	51.33	45.3	65.59	95
180	Alphagaurd	–	–	–	–	–	–
	RAD7	–	–	–	–	–	–
	Raduet	75	95	64	57	–	73
	NRPB	77	80	58	66	–	69
	Simulation	110.12	121.21	52.3	46.23	59.17	79

## Conclusion

The minimum standard ventilation rate for dwellings is important not only to ensure the health and comfort of dwellers but also to eliminate and dilute the dominant pollutants. Recently, the CFD method has drawn attention to the prediction and visualization of the distribution pattern of radon and thoron concentrations in confined areas. The purpose of this survey is to estimate the radon concentration at different ventilation rates for a typical room by using the CFD technique before comparing and validating the CFD results with analytical calculations and experimental measurements. This study applied both an experimental and CFD model (using the commercially available CFD software ANSYS Fluent 2020 R1 based on the FVM method) to investigate the radon dispersion under typical indoor ventilation (natural ventilation) as well as open- and closed-door scenarios. The calculations were validated by comparing the CFD results with active measurements taken by the AlphaGUARD and RAD7 radon monitors as well as passive measurements recorded by NRPB and RADUET detectors based on CR-39. These results would be useful for the organizations and authorities to have a picture of critical point of higher indoor radon concentration which should take into consideration for dose assessment.

By assuming an air exchange rate of 1 h^−1^ to comply with ventilation requirements, the radon concentrations in the middle of the room (in both scenarios) were low and the average radon concentrations from the CFD simulations were 70.21 and 66.25 Bq m^−3^ in the closed- and open-door scenarios, respectively. The difference between the results of the analytical calculations and CFD simulations were found to be approximately 11% and 5% in the open- and closed-door scenarios, respectively. The measured radon concentrations recorded by the active measurements were also in good agreement with the CFD results, e.g., with a relative deviation of approximately 7% and 2% according to the AlphaGUARD and RAD7 radon monitors at a height of 1.0 m above the ground in the open-door scenario. Moreover, the maximum relative deviation of 39% was recorded by the NRPB detectors at a height of 20 cm above the ground in the closed-door scenario. The highest radon concentrations were detected in close proximity to the floor and upper wall around the inlet which was reduced by increasing the air exchange rate, while the lowest values were observed close to the inlet and front wall. On the basis of these results, it can be concluded that these trends are due to the air velocity profile. The simulation results revealed that the air velocity distribution inside the room plays a major role with regard to the distribution of the indoor radon concentration. The results also demonstrate that CFD modelling is capable of predicting the indoor distribution of radon gas. Finally, regarding mitigation of radon, the best ways are^26–28^: (1) as shown in the simulation, increase air flow in the confined area by opening windows and using fans and vents to circulate air; (2) Sealing the cracks in floors and walls with plaster, caulk, or other materials designed for this purpose.

## Data Availability

The data sets used and/or analyzed during the current study are available from the corresponding author on request.
